# Chinese Unknown Word Recognition for PCFG-LA Parsing

**DOI:** 10.1155/2014/959328

**Published:** 2014-04-09

**Authors:** Qiuping Huang, Liangye He, Derek F. Wong, Lidia S. Chao

**Affiliations:** NLP^2^CT Laboratory, Department of Computer and Information Science, University of Macau, Macau

## Abstract

This paper investigates the recognition of unknown words in Chinese parsing. Two methods are proposed to handle this problem. One is the modification of a character-based model. We model the emission probability of an unknown word using the first and last characters in the word. It aims to reduce the POS tag ambiguities of unknown words to improve the parsing performance. In addition, a novel method, using graph-based semisupervised learning (SSL), is proposed to improve the syntax parsing of unknown words. Its goal is to discover additional lexical knowledge from a large amount of unlabeled data to help the syntax parsing. The method is mainly to propagate lexical emission probabilities to unknown words by building the similarity graphs over the words of labeled and unlabeled data. The derived distributions are incorporated into the parsing process. The proposed methods are effective in dealing with the unknown words to improve the parsing. Empirical results for Penn Chinese Treebank and TCT Treebank revealed its effectiveness.

## 1. Introduction

Parsing plays an important role in natural language processing. In recent years, Chinese parsing has received a great deal of attention, and lots of researchers have presented many of Chinese parsing models [1–3]. Nevertheless, as pointed out in [4], the improved performance around 84% *F*-measure that still falls far short of performance on English. This leaves a large space for the further improvement of Chinese parsing.

As far as we know, there is a large portion of fixed errors coming from unknown words in Chinese parsing. Therefore, a robust parser must have a mechanism of processing unknown words, where it discovers the POS tag and features information about unknown words during parsing. A number of researches design hand-crafted rules or make use of rich morphological features to handle them. It is well known that Chinese words tend to have greater POS tag ambiguities than English and the morphological properties of Chinese words are complicated to be predicted of POS type for unknown words. For this reason, we present a more effective character-based model to handle unknown words according to [5]. The method mainly used an exponential function to represent the distance between the head character and other characters in an unknown word and use the geometric average to estimate the emission probability of it. Besides, we present a novel method to deal with unknown words by using graph-based semisupervised learning. Graph-based label propagation methods have made a remarkable improvement in several natural language processing tasks, for example, knowledge acquisition [6], Chinese word segmentation, POS tagging [7], and Chunking [8]. In this paper, this approach is used to propagate POS tag and derive the emission probabilities to the large amount of unlabeled data by utilizing the limited resource (e.g., POS information from the labeled data, i.e., Penn Chinese Treebank and lexical emission probability learned by the PCFG-LA model). Then the derived unlabeled information generated by graph-based knowledge will be incorporated into the parser. In fact, this method explores a new way to exploit the use of unlabeled data to strengthen the supervised model in parsing, building on the technique presented in [9], which strengthens the lexical model by using a graph-based lexical expansion approach.

This paper is structured as follows.  Section 2 reviews the background of the lexical model in the Berkeley PCFG-LA model.  Section 3 describes the modification of the character-based model in this study.  Section 4 presents the details of the proposed approach based on graph-based semisupervised learning.  Section 5 makes comparisons with other unknown word recognition models. Experiments setup and result analysis are reported in Section 6. The last section draws the conclusion.

## 2. Background

The Berkeley parser [3, 10] is an efficient and effective parser that introduces latent annotations to learn high accurate context-free grammar (CFG) directly from a Treebank. Nevertheless, the lexical model of grammar is not well designed to effectively handle the out-of-vocabulary (OOV) words (a.k.a. unknown words) universally and the OOV model of Berkeley parser has proved to be more suitable for English in [4, 11]. The built-in treatment to unseen words of Berkeley parser can be concluded as utilizing the estimation of rare words (in the newest version of Berkeley parser, words with frequency less than 10 will be regarded as rare words acquiescently) to reflect the appearance likelihood of OOV words.

In order to get the more refined and accurate grammar, Petrov et al. [10] developed a simple split-merge-smooth training procedure. In order to counteract overfitting problem, they introduced a linear smoothing method to smooth the lexical emission probabilities:

(1) $$\begin{array}{c} \bar{P}=\frac{1}{\left|t\right|}\underset{x}{\sum }P_{\theta }\left(wt_{x}\right), \end{array}$$

(2) $$\begin{array}{c} P_{\theta }\left(w\;|\;t_{x}\right)⟵\varepsilon \bar{P}+\left(1-\varepsilon \right)P_{\theta }\left(w\;|\;t_{x}\right), \end{array}$$

where |*t*| denotes the number of latent tags from *t* and *t*_*x*_ means a set of latent subcategories {*t*_*x*_ | *x* = 1,…, |*t*|}. In (1), *θ* is the model parameters which can be optimized by EM-algorithm. In (2), *ε* is a smoothing parameter.

Since the lexical model can only generate words observed in the training data, a separate module is needed to handle the OOV words that appear in the test sentences. There are two ways to estimate an OOV word *w* based on a specific latent tag *t*_*x*_. One is assigning the probability of generating rare words in the training data by *t*_*x*_  :  *P*_*θ*_(*rare* | *t*_*x*_); another is, suggested by the Berkeley parser as* Sophisticated Lexicon*, to calculate the emission probability through analysing the morphological features of the OOV words. In the Berkeley parser, English words are classified into a set of signatures based on the presence of characters, especially on a list of inherent suffixes (e.g.,* -ed, -ing*); then the estimation of *w*/*t*_*x*_ pair is

(3) $$\begin{array}{c} P_{\theta }\left(w\;|\;t_{x}\right)\propto P_{\theta }\left(s\;|\;t_{x}\right), \end{array}$$

where *s* is the OOV signature for *w* and *P*_*θ*_(*s* | *t*_*x*_) is computed by *e*_*t*_*x*_,*s*_/*e*_*t*_*x*__.

Nevertheless, the features applied to Chinese word are simpler than English. Only the last character of word will be taken into account in estimating emission probabilities of rare word. Before applying such model, OOV words will be checked if they belong to temporal noun (NT) (by checking if the word contains characters like “*年*” (year), “*月*” (month), or “*日*” “*号*” (day)), cardinal number (CD) (by checking if the word contains character of number), ordinal number (OD) (by checking if the word contains character, such as “*第*”), or proper noun (NR) (by checking if the word contains character, such as “•”) preferentially.

## 3. Modification of Character-Based Model

In this study, we make the modification deriving from two reasons. First of all, the Berkeley parser is adequate for English and only a limited number of classes of unknown words are handled for Chinese. In parsing phase, if the unknown words belong to the categories of digit or date, the Berkeley parser has some inbuilt ability to handle them. For words excluded from these classes, the parser ignores character level information and decides these word categories only on the rare word POS tag statistics. Let *t* denote the tag, and let *w* denote the word. The model for estimation of the unknown word probability somehow can be written in this format: *P*(*w* | *t*). Besides, we know that the Chinese words formation process can be quite complex differing from the English process. The characters in any position (prefix, infix, or suffix) can be predictive of the POS type for Chinese word. Therefore, in our study, we employ a more effective method, which is similar to but more detailed than the work of Huang et al. [5], to compute the word emission probability to build up our new Chinese unknown word model. The geometric average of the emission probability of the characters in the word is applied. We use *c*_*k*_ to denote *k*th character in the word. Since some of the characters in *w*_*i*_ may not have appeared in any word tagged as *t*_*i*_ in that context in the training data, only characters that are mentioned in the context are included in the estimate of the geometric average; then *P*(*c*_*k*_ | *t*_*i*_) is achieved:  

(4) $$\begin{array}{c} P\left(w_{i}\;|\;t_{i}\right)=\sqrt[{\sum \theta }]{\underset{c_{k\in w_{i},\;\;p(c_{k}\;|\;t_{i_{k}})\neq 0}}{\prod }P{\left(c_{k}\;|\;t_{i_{k}}\right)}^{\theta_{k}},} \end{array}$$

where

(5) $$\begin{array}{c} n=\left|\left\{c_{k}\in w_{i}\;|\;P\left(c_{k}\;|\;t_{i_{k}}\right)\neq 0\right\}\right|,\\ \theta_{k}=\exp\left(-\text{dis}\left(c_{k}\right)\right). \end{array}$$

In (4), we use *θ*_*k*_ to assign a weight to the emission probability of each character *c*_*k*_. We determine the head character and use an exponential function to represent the distance between the head character and another character. In our experiment, we use the first character and the last character as the head character, respectively, and try out which position in a Chinese word is most important.

As we can see in Table 1, the modified character-based model improves performance on both recall and precision compared to the Berkeley baseline model when evaluated on TCT [12].

## 4. Graph-Based OOV Model

### 4.1. The Background of Graph-Based Label Propagation

Graph-based label propagation, a critical subclass of semisupervised learning (SSL), has been widely used and shown to outperform other SSL methods [13]. Most of these algorithms are transductive (transductive learning is used to contrast inductive learning; a learner is transductive if he only works on the labeled and unlabeled training data and cannot handle unseen data) in nature, so they cannot be used to predict an unseen test example in the future [14]. Typically, graph-based label propagation algorithms are run in two main steps: graph construction and label propagation. The graph construction provides a natural way to represent data in a variety of target domains. One constructs a graph whose vertices consist of labeled and unlabeled data. Pairs of vertices are connected by weighted edges which encode the degree to which they are expected to have the same label [15]. The great importance of graph construction methods leads to a number of graph construction algorithms in the past years. Popular graph construction methods include *k*-nearest neighbors (*k-*NN), e-neighborhood, and local reconstruction. In this paper, the *k-*NN method is used to construct the graph. Besides, label propagation operates on the constructed graph. Its primary objective is to propagate labels from a few labeled vertices to the entire graph by optimizing a loss function based on the constraints or properties derived from the graph, for example, smoothness [15–17] or sparsity [18]. State-of-the-art label propagation algorithms include LP-ZGL [15], Adsorption [19], MAD [17], and Sparsity-Inducing Penalties [18]. The Sparsity-Inducing Penalties algorithm is used in this study.

### 4.2. The Proposed Method

The emphasis of this paper is on presenting a method to recognize Chinese unknown words by using two different kinds of data sources, for example, labeled texts and unlabeled texts, to construct a specific similarity graph. In essence, this problem can be treated as incorporating gainful information, for example, prior knowledge or label constraints, of unlabeled data into the supervised model. In our approach, we employ a transductive graph-based label propagation method to achieve such gainful information; for example, label distributions are inferred from a similarity graph constructed over labeled and unlabeled data. Then, the derived label distributions are regarded as “soft evidence” to augment the parsing of Chinese unknown words based on a new learning objective function. The algorithm contains the following two stages (see Algorithm  1). Firstly, given labeled data and unlabeled data, that is, *T*_*l*_ = {*w*_*i*_}_*i*=1_^*l*^ with *l* labeled words and *T*_*u*_ = {*w*_*i*_}_*i*=*l*+1_^*l*+*u*^ with *u* unlabeled words, a specific similarity graph {*G*} representing *T*_*l*_ and *T*_*u*_ is constructed (POS tag graph). In this stage, we construct one graph over all of labeled data and unlabeled data and propagate one POS tag for each unlabeled word (see Section 4.2.1). Secondly, probabilities of latent tag *P*_*θ*_(*w* | *t*_*x*_) are estimated subsequently. In this application, we will generate *N* graphs, where *N* stands for the number of POS type; each graph is aimed at propagating latent tag for the unlabeled words in their most probable POS tag, which can be determined from the graph in first stage (see Section 4.2.2).

#### 4.2.1. Assigning POS Tags to Unlabeled Words

In this stage (corresponding to procedures 1–3 in Algorithm 1), the common practice is to construct a similarity graph for the labeled data and unlabeled data and aims at assigning a POS tag to unlabeled data in a vertex constructing and label propagation tradition. The effect of the label propagation depends heavily on the quality of the graph. Thus graph construction plays a central role in graph-based label propagation [15].

In this stage, we represent vertices by all of the word trigrams with occurrences in labeled and unlabeled sentences to construct the first graph. The graph construction is nontrivial. As Das and Petrov [20] mentioned, taking individual words as the vertices would result in various ambiguities and the similarity measurement is still challenging. Therefore, in this paper, we follow the same intuitions of graph construction from [21] by using trigram and the objective focuses on the center word in each vertex. Formally, we are given a set of labeled texts *T*_*l*_ = {*w*_*i*_}_*i*=1_^*l*^ and a set of unlabeled texts *T*_*u*_ = {*w*_*i*_}_*i*=*l*+1_^*l*+*u*^. The goal is to form an undirected weighted graph *G* = (*V*, *E*), in which *V* is the set of vertices, which covers all trigrams extracted from *T*_*l*_ and *T*_*u*_. Here *V* = *V*_*l*_ ∪ *V*_*u*_, where *V*_*l*_ refers to trigrams that occur at least once in labeled data and *V*_*u*_ refers to trigrams that occur only in the unlabeled data. The edge *E* ∈ *V* × *V*. In our case, we make use of the *k*-nearest neighbors (*k-*NN) (*k* = 5) method to construct the graph and the edge weights are measured by a symmetric similarity function as follows:

(6) $$\begin{array}{c} w_{i,j}=\{\begin{array}{cc} \text{sim}\left(x_{i},x_{j}\right), & \text{if}\;j\in K\left(i\right)\;\;\text{or }i\in K\left(j\right)\\ 0, & \text{otherwise,} \end{array} \end{array}$$

where *x* denotes one vertex in the graph, *K*(*i*) is the *k*-nearest neighbors of *x*_*i*_ (|*K*(*i*) = *k*, ∀*i*|), and sim(*x*_*i*_, *x*_*j*_) is a symmetric similarity measure between two vertices. The similarity function is computed based on the cooccurrence statistics over the features shown in Table 2.

To induce label distributions of unlabeled word from labeled vertices to entire graph, the label propagation algorithm Sparsity-Inducing Penalties (Sparsity) proposed by [18] is employed in this study. The following convex objective function is optimized in our case:

(7) argmin⁡q⁡ ∑j=1l||qj−rj||2+μ∑i=1,k∈N(i)mwik||qi−qk||2+λ∑i=1mqi2s.t.      q≥0, ∀i∈V, ||qi||1=1,

where *r*_*j*_ denotes empirical label distributions of labeled vertices and *q*_*i*_ denotes unnormalized estimate measures in every vertex. The *w*_*ik*_ refers to the similarity between trigram *i* and trigram *k*, and *N*(*i*) is a set of neighbors of trigram *i*. *μ* and *λ* are two hyperparameters. The squared-loss (e.g., ||*p*||^2^ = ∑_*y*_ *p* ^2^(*y*), which can be seen as a multiclass extension of the quadratic cost criterion Bengio et al. [22] or as a variant of one of the objectives in Zhu et al. [23]) criterion is used to formulate the objective function. The first term in (7) is the seed match loss which penalizes *q*_*j*_ if they go too far away from the empirical labeled distribution *r*_*j*_. The second term is the edge smoothness loss that requires *q*_*i*_ to be smoothed with respect to the graph, such that two vertices connected by an edge with high weight should be assigned similar labels. The final term is a regularizer to incorporate the prior knowledge, for example, uniform distributions used in [20, 21].

The estimated label distribution *q*_*i*_ in (7) is relaxed to be unnormalized, which simplifies the optimization. Therefore, the objective function in (7) can be optimized by LBFGS-B [24], a generic quasi-Newton gradient-based optimizer.

Mathematically, the problem of label propagation is to get the optimal emission label distribution *q*_*i*_ of every labeled vertex. Integrating the similarity between every two vertices, we can project the most probable POS (selection from the *q*_*i*_) tag to the unlabeled words.

Through the construction of similarity graph and propagation of labels in this stage, each unlabeled word will get a POS tag.

#### 4.2.2. Generating Latent Tag and Emission Probability to Unlabeled Words

In this stage (corresponding to procedures 4–7 in Algorithm 1), we mainly construct another type of graph {*g*} to generate latent tag and emission probability to unlabeled words. As mentioned, each unlabeled word gets only one POS tag in stage one. Consequently, we build a graph for each type POS tag, respectively, in order to obtain an optimal emission probability distribution for each unlabeled word at this stage. When constructing the similarity graph, each vertex represents a word instead of a trigram because we only need to consider this word's latent tags and emission probability distribution based on its POS tag generated in stage one. The graph construction and label propagation procedures are similar to those of the previous stage. It is worth noting that ||*q*_*i*_||_1_ ≠ 1 in (6) which differs from the previous stage. The emission distribution *q*_*i*_ is generated from all possible vertices with the same POS tag in a similarity graph instead of all of possible POS types of a vertex. Finally, the label distributions can be propagated to the unlabeled words, and the label distribution content is the same as the Berkeley lexicon (contains the respective rule scores and words) trained by Berkeley parser.

### 4.3. Incorporation

After the former steps, we can get a lexicon of unlabeled words with label distribution. The lexicon is treated as an OOV lexicon which covers most of OOV words that appear in testing data but not in the training data in our system. Then this OOV lexicon should be incorporated into the Berkeley parser. Our strategy of insertion is that when an OOV word is detected, it should be firstly examined if the OOV lexicon contains such word; then corresponding estimation will be used; otherwise, the built-in OOV word model (mentioned in Section 2) will be used. During the parameter tuning phase, we try to use linear incorporation to inspect the impact of our OOV model on the whole parsing model:

(8) $$\begin{array}{c} \alpha \theta_{o}+\left(1-\alpha \right)\theta_{b}\;\text{s.t.}\;\;0\leq \alpha \leq 1, \end{array}$$

where *θ*_*o*_, *θ*_*b*_ denote the estimation generated by our proposed OOV model and the Berkeley model, respectively.

## 5. Comparison with Other OOV Recognition Models

The proposed approaches in this paper differ from previous OOV recognition models. Collins [25] assigned the UNKNOWN token to unknown words, and any tag/word pairs not seen in training data would give a zero of estimation. Klein and Manning [1] designed a simple method to estimate the emission probability of an unknown word based on how likely it is that the subcategory generates a rare word in the training data. For each of these categories, they took the maximum-likelihood estimation of *P*(*tag* | *wordclass*) and add a parameter *k* to smooth and accommodate unknown words. In [10], they mainly utilized the estimation of rare words to reflect the appearance likelihood of OOV words and the details of the method have been mentioned in Section 2. Inspired by [11, 13], we improved Chinese unknown word parsing performance by using the geometric average of emission probabilities of first character and last character in the word. Furthermore, differing from these concerns, we make use of a new perspective to employ unlabeled data to augment the supervised model and to handle the OOV word by graph-based semisupervised learning. Our emphasis is to learn the semisupervised model by smoothing the label distributions that are derived from a specific graph constructed with labeled and unlabeled data. Though graph-based knowledge, the OOV label distribution can be generated. It is worth noting that the selection of unlabeled data should cover OOV words as much as possible because this approach is mainly used to assign a POS tag and emission probabilities to each unlabeled data according to the similarity between any two vertices in a graph constructing among labeled data and unlabeled data. If all of OOV words are found in the unlabeled data, then each OOV word would be recognized by our model. When we construct a graph where a portion of vertices correspond to labeled instances, and the rest is unlabeled. Pairs of vertices are connected by a weighted edge denoting the similarity between the pair. In this process, optimization of a loss function based on smoothness properties of the graph is performed to propagate labels from the labeled vertices to the unlabeled ones. Overall, this method differs in three important aspects: firstly, the existing resource (e.g., annotated Treebank and the latent variable grammars induced by Berkeley parsing model) is well utilized; secondly, the training procedure is simpler than that of [26]; thirdly, the derived label information from the graph is smoothed into the model by optimizing a modified objective function.

## 6. Experiment

### 6.1. Data Sets

The experimental data are mainly taken from the Chinese Treebank (CTB-5.0) and TCT Treebank [12]. CTB-5.0 consists of about 507,222 words of annotated and parsed text from newswire. It is a segmented, POS tagged, and fully bracketed corpus. TCT contains 17,558 sentences and about 480,000 Chinese words. The Treebank uses a double-tagging annotation scheme, for example, (zj-XX (fj-LS (dj (nP *江泽民*) (v *指出*)) (dj-RT (wP  ,) (dj (vp (v *搞好*) (np (n *物价*) (n *工作*))) (vp (dD *极*) (vp (v *为*) (a *重要*)))))) (wE  *。*)). In this sentence,* zj, dj, np*, and so forth are the syntactic tags and LS and RT are grammatical relation tags. In order to adopt the same evaluation metric with the CTB Treebank, we remove the grammatical relation tags and only retain the syntactic tags in the experimental data. Besides, the Peking University Corpus in Second International Chinese Word Segmentation Bakeoff (http://www.sighan.org/bakeoff2005/) is utilized as unlabeled data *T*_*u*_ for our graph-based OOV model. The corresponding statistic information on these two Treebanks is shown in Tables 3 and 4, respectively. EVALB [27] is used for the evaluation.

### 6.2. Results and Analysis

We firstly run the experiment on the TCT Treebank with the character-based model. The model has an overall POS tags accuracy of 94.80%, which is slightly higher than the Berkeley baseline model. This may be because the proposed model cannot well extract the features from the unknown words to improve the POS tagging. However, the parsing result is 82.83% that has a great improvement over the baseline accuracy of 80.98%. The detailed result is showed in Table 5.

Next, we use the CTB-5.0 Treebank and TCT Treebank to do the experiments in the graph-based OOV model separately. In our model, the parameter *α* is smoothed to accommodate OOV model used in (8). When *α* = 0, the model uses only the lexical model estimation. While *α* = 1, it uses only the graph-based OOV model prediction of words. It is interesting to note that the combination model results in improvement over the baseline lexical model in terms of *F*-score and OOV accuracy on CTB-5.0 and TCT. When *α* = 1, the estimation performs the best result. This strongly reveals that the knowledge derived from the similarity graph does effectively strengthen the model. Tables 6 and 7 demonstrate the parsing results on the testing set in these two Treebanks. The best improvements in POS tagging and parsing are 0.89% and 0.65%, respectively, in CTB Treebank. In the TCT Treebank, the OOV model contributes to 1.04% and 0.33% improvement on the accuracy of POS tagging and syntax parsing. From the result, we can see that our model outperforms the baseline by incorporating unlabeled data to boost the supervised model. The main reason is that unlabeled data lack information; we use transductive graph-based label distributions derived from labeled data. The derived label information is considered as prior knowledge relative to unlabeled data, thereby enriching the training data. Most importantly, the similarity graph can also be allowed to propagate the label distributions for unknown words to augment their parsing.

## 7. Conclusion

In this paper, we try to use the modified character-based model to improve the performance of a PCFG-LA parser. Simultaneously, we show for the first time that the graph-based semisupervised learning is able to improve the performance of a PCFG-LA parser on OOV words. The approach mainly uses a *k*-nearest neighbor algorithm to construct a similarity graph based on labeled and unlabeled data and then incorporates the graph knowledge into the Berkeley parser. Experimental comparisons on the CTB and TCT corpus indicate that the proposed approaches are better than the baseline model.

## Figures and Tables

**Algorithm 1:**
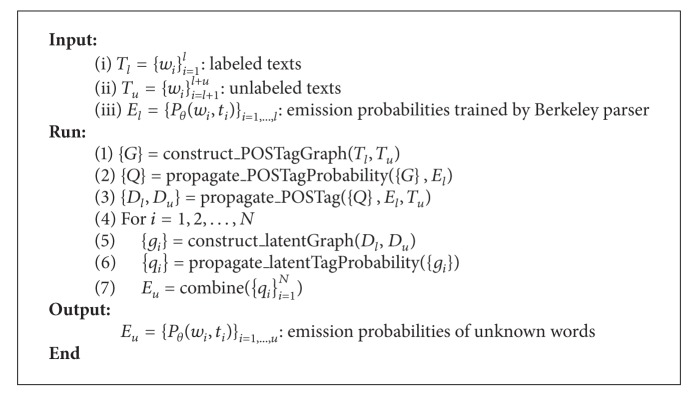
Words label propagation algorithm.

**Table 1:** The effect of the character-based model on TCT.

	Length	*R*	*P*	*F*
Baseline	All	80.97	80.99	80.98
	≤40	83.56	83.55	83.55

Character-based	All	82.76	82.47	82.83
	≤40	84.96	85.08	85.02

**Table 2:** Features employed to measure the similarity between two vertices, in a given text example “*他非常专业*” (I am very happy), where the trigram is “*非常专*”.

Feature	Example
Trigram + Context	*他非* *常专* *业*
Trigram	*非* *常专*
Left Context	*他非*
Right Context	*专业*
Center Word	*常*
Left Word + Right Word	*非专*
Left Word + Right Context	*非* *专业*
Left Context + Right Word	*他非* *专*

**Table 3:** The statistics summary of data in CTB-5.0.

	Train	Unlabeled	Dev	Test
#Sentence	17,785	19,075	352	348
#Word	485,230	1,110,947	6,821	8,008
#OOV	—	—	382	263

**Table 4:** The statistics summary of data in TCT.

	Train	Unlabeled	Dev	Test
#Sentence	14,045	19,075	1,755	1,758
#Word	377,303	1,110,947	47,836	48,449
#OOV	—	—	1,928	1,916

**Table 5:** POS and parsing accuracy on TCT in character-based model.

	Length	*R*	*P*	*F*	POS
Baseline	All	80.97	80.99	80.98	94.51
	≤40	83.56	83.55	83.55	94.56

TCT	All	82.76	82.47	82.83	94.80
	≤40	84.96	85.08	85.02	94.76

**Table 6:** POS and parsing accuracy on CTB in graph-based OOV model.

	Length	*R*	*P*	*F*	POS
Baseline	All	78.34	82.68	80.45	94.88
	≤40	81.78	85.63	83.66	95.58

CTB	All	78.90	83.20	80.99	95.77
	≤40	82.38	86.34	84.31	96.31

**Table 7:** POS and parsing accuracy on TCT in graph-based OOV model.

	Length	*R*	*P*	*F*	POS
Baseline	All	80.97	80.99	80.98	94.51
	≤40	83.56	83.55	83.55	94.56

TCT	All	81.30	81.32	81.31	95.51
	≤40	83.92	83.91	83.92	95.60
